# 

**Published:** 1875-03

**Authors:** 


### TABLE OF CONTENTS.

ORIGINAL COMMUNICATIONS.

On Rattlesnake Poison, Rabies Canina, etc. By Alban S. Payne, M.D., of Va. 129

SELECTIONS.

Quinine in Suppuration. By Douglas Morton, A.M., M.D.. ................ 146
Gum-Cutting. By Charles E. Buckingham, M.D..... .......... ........ 149
On the Employment of Tepid Baths in Diseases of the Chest, especially in Pulmonary Phthisis.... ......... .. ........................ .... 152
A New Mode of Taxis for Strangulated Hernia. By Augustus F. Erich, M.D. 154
The Advantages and Disadvantages of Esmarch's Bloodless Method. ....... 157
Treatment of Spermatorrhœa by a Double Truss.......................... 159
Case of Amalgamated Placenta.......................... ...... ....... 161
The Rectum in its Relation to Uterine Disease. By Arthur W. Edis, M.D.... 163

EXTRACTS AND GLEANINGS.

The Dispersion of Tumors by Puncture—164. Case of Chronic Arsenic Poisoning—165. Dysentery Cured without Opium; Aconite in Headache—166. Bromide of Camphor; Nitrite of Amyl and Belladonna in Dysmenorrhœa—167. Fissures in Ano Treated with Iodoform; Physician as a Witness—168. The Treatment of Fistulous Sinuses by the Elastic Ligature; Typhoid Fever—169. The Extraction of Foreign Bodies from the Ear; Diabetes—170. Treatment of Delirium Tremens; Turpentine an Antidote for Phosphorus—171. Treatment of Catarrhal Jaundice by Electricity; Horse-Hair for Sutures—172. Bulk of Salts in Solution; A Strange Story; Soft Chancres—173. New Liniment for Scabies; The Hydrate of Chloral to Arrest Putrefaction and Fermentation; Pityriasis Versicolor—174. Antagonism between Chloral Hydrate and Calabar Bean; Nasal Catarrh; Treatment of Epitaxis—175. Deafness Cured by the Extraction of Teeth; Treatment of Persistent Neuralgia; Improved Glycerin Cement—176. Epilepsy cured by an Excision of a Neuroma; Lime Glycerin for Burns—177. Mr. Erichsen on American Surgery; Bromine in Pertussis; Warts—178. Cæsarean Section for both Mother and Child; Copaiba in Dropsy—179. Differential Diagnosis between the Red Blood Corpuscle of Human and Certain of the Lower Animals; Toothache Drops; A Cure for Bright's Disease—180. Acidulated Gargles in Typhoid Fever; Preparations of Sponge Tents; Toothache Drops—181. Ailanthus Glandulosa in Dysentery; A Remedy for Toothache; Guaiacum in Acute Diseases of the Throat—182. Hyperdermic Injection of Ergot in Post-partum Hemorrhage; Preparation of Koumiss; Cardiac Œdema—183. Glycerin-Sichel; Catheter Impacted in the Female Pelvis; A Fly of Africa—184.

EDITORIAL AND MISCELLANEOUS.

Medical Association of Georgia.......... .......................... 185
Query .............................................................. 186
American Medical Association ... .................................. 189
Action of the Middle Georgia Medical Society........................ 190
Dr. A. S. Payne.................................... .. ........... 191
Tribute of Respect; List of Cash Payments to the Record ................ 192

